# Noncoding RNAs as sensors of tumor microenvironmental stress

**DOI:** 10.1186/s13046-022-02433-y

**Published:** 2022-07-16

**Authors:** Yue Lv, Yinghao Lv, Zhen Wang, Kefei Yuan, Yong Zeng

**Affiliations:** 1 Department of Liver Surgery & Liver Transplantation, State Key Laboratory of Biotherapy and Cancer Center, West China Hospital, Sichuan University and Collaborative Innovation Center of Biotherapy, Chengdu, China; 2 Laboratory of Liver Surgery, West China Hospital, Sichuan University, Chengdu, 610041 China

**Keywords:** LncRNAs, CircRNAs, Hypoxia, Inflammation, Nutrient deprivation

## Abstract

The tumor microenvironment (TME) has been demonstrated to modulate the biological behavior of tumors intensively. Multiple stress conditions are widely observed in the TME of many cancer types, such as hypoxia, inflammation, and nutrient deprivation. Recently, accumulating evidence demonstrates that the expression levels of noncoding RNAs (ncRNAs) are dramatically altered by TME stress, and the dysregulated ncRNAs can in turn regulate tumor cell proliferation, metastasis, and drug resistance. In this review, we elaborate on the signal transduction pathways or epigenetic pathways by which hypoxia-inducible factors (HIFs), inflammatory factors, and nutrient deprivation in TME regulate ncRNAs, and highlight the pivotal roles of TME stress-related ncRNAs in tumors. This helps to clarify the molecular regulatory networks between TME and ncRNAs, which may provide potential targets for cancer therapy.

**Supplementary Information:**

The online version contains supplementary material available at 10.1186/s13046-022-02433-y.

## Background

Until the 1980s, cancer research was dominated by a tumor-centric view assuming that mutations in oncogenes and tumor suppressor genes were responsible for the whole processes of cancer development and progression before. However, with the continuous deepening of the research, scientists have shifted their focus from cancer cells to their surrounding microenvironment [1]. The tumor microenvironment (TME) mainly consists of diverse cellular and non-cellular constituents. The cellular constituents comprise endothelial cells, cancer-associated fibroblasts (CAFs), tumor-associated macrophages (TAMs), myeloid-derived suppressor cells (MDSCs), lymphocytes, antigen-presenting cells, pericytes, and neoplastic cells. Non-cellular constituents include extracellular matrix as well as secreted products of TME cells, such as pro-inflammatory cytokines, chemokines, growth factors, and metabolites [2]. Undergoing a series of dynamic alterations, the physical and chemical characteristics of TME become different from those of normal tissues. Most strikingly, various stress conditions emerge in TME, such as low oxygen tension, acidosis, inflammation, nutrient deprivation, oxidative stress, and endoplasmic reticulum stress. Stress conditions of TME exhibit strong correlations with cancer malignancy by playing critical roles in tumor growth, metastasis, and therapy resistance [2–6].

Large-scale genomic sequencing revealed that less than 2% of human genome transcripts encode proteins, whereas the vast majority can be transcribed into different classes of noncoding RNAs. Noncoding RNAs (ncRNAs) are defined as transcripts with minimal or no protein-coding capacity. NcRNAs can be roughly classified into two major categories based on their molecular length, including small ncRNAs (sncRNAs) (< 200nt in length) and long ncRNAs (lncRNAs) (> 200 nt in length). The sncRNAs consist of microRNAs (miRNAs), small interfering RNAs (siRNAs), small nucleolar RNAs (snoRNAs), small nuclear RNAs (snRNAs); transfer RNA-derived small RNAs (tsRNAs), PIWI-interacting RNAs (piRNAs), etc. Notably, lncRNAs constitute a large portion of noncoding transcripts of the human genome [7, 8]. NcRNAs serve as functional regulatory molecules that mediate gene transcription, chromatin remodeling, post-transcriptional modification, and signal transduction. Numerous studies have identified ncRNAs as either oncogenic drivers or tumor suppressors that are implicated in diverse cellular processes, including tumor cell proliferation, metabolism, apoptosis, metastasis, immune evasion, and resistance to therapies, etc. [7, 9]. With technology advances like high-throughput transcriptome sequencing and RNA-microarray, an impressive number of ncRNAs were identified to be differentially expressed in cancer in response to different TME stresses [10–14]. Consequently, elucidation of the complicated interactions between TME stress and ncRNAs in the process of tumorigenesis and development has become a research hotspot.

In this review, we systematically summarize the diverse molecular mechanisms by which various TME stresses dysregulate ncRNA expression, and discuss how these stress-responsive ncRNAs regulate tumorigenesis and cancer progression.

## NcRNAs regulated by hypoxia

**Fig. 1:**
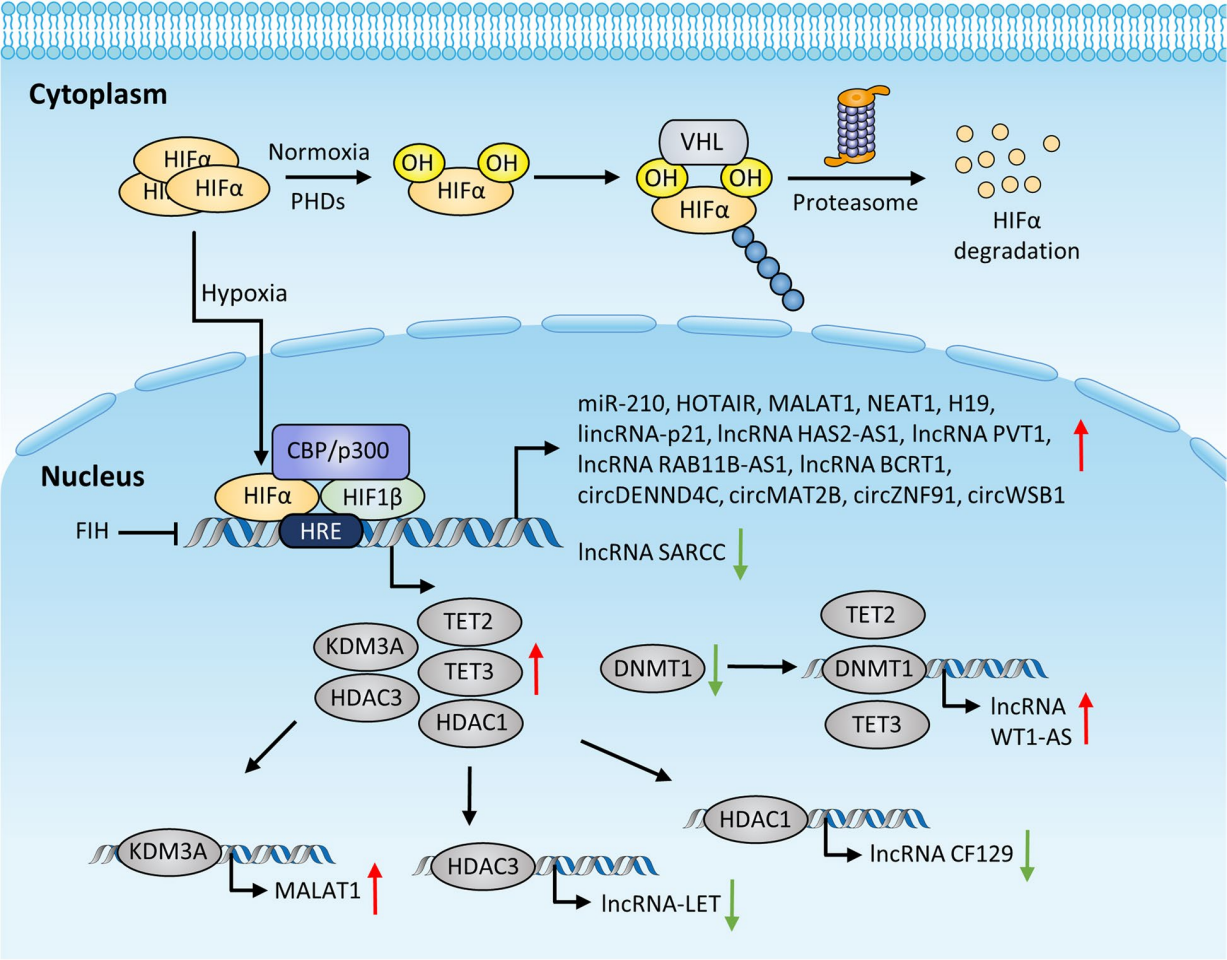
The graphical representation of the HIFs-mediated regulatory mechanisms of ncRNA transcription upon hypoxia in TME. Under normoxia, HIFα subunit is hydroxylated by PHDs. Upon hydroxylation, HIFα interacts and binds to the VHL protein, which acts as a ubiquitin E3 ligase, eventually leading to proteasomal degradation of HIFα. FIH hydroxylates HIFα and halts its binding to CBP/p300. Upon hypoxia, oxygen depletion inhibits the hydroxylation of PHDs and FIH to promote HIFα stabilization and nucleus translocation. HIFα dimerizes with HIF-1β, recruits coactivator CBP/p300, and then binds to the HREs of target genes to regulate the transcription of various ncRNAs. Additionally, in hypoxic TME, HIFs upregulate multiple chromatin-modifying enzymes such as HDAC1/HDAC3/KDM3A/TET2/TET3 or downregulate DNMT1, thereby affecting ncRNA transcription via chromatin modification

Intratumoral hypoxia (0.5–2% oxygen), a common hallmark in TME, originates from a mismatch between insufficient oxygen supply caused by ineffective vasculature and elevated metabolic demands from rapid cancer cell proliferation. Cancer cells sense a decline in oxygen level and elicit multiple genomic alterations and adaptive cellular responses, enabling themselves to adapt to low oxygen availability, which further promotes cancer initiation and progression [4]. Of note, emerging evidence has indicated that a vast variety of ncRNAs (e.g., miRNA, lncRNA, circRNA, and piRNA) are differentially expressed in response to hypoxia and exert crucial roles in cancer development [10, 15]. Nevertheless, the diverse mechanisms whereby hypoxia modulates ncRNAs have rarely been systematically discussed. Hypoxia-inducible factors (HIFs), as central regulators for detecting and adapting to fluctuations in oxygen level, are known to orchestrate hypoxia-responsive gene expression in cancer [16]. Herein, based on the relevance to transcription factor HIFs, we simply categorize the regulatory mechanisms of ncRNAs upon hypoxia into regulations with the involvement of HIFs (HIFs-dependent), or regulations without the involvement of HIFs (HIFs-independent). The detailed modes of regulation and functions of these hypoxia-responsive ncRNAs in cancer are summarized in the section below (Table 1, Fig. 1).

**Table 1:** Hypoxia-regulated ncRNAs and their roles during cancer progression

NcRNAs	Expression upon hypoxia	Regulatory mechanisms	Functions	Cancer types	References
miRNA-210	Up-regulated	HIF-1α/HIF-2α/p53/NF-κB/Oct-4/PPARγ-dependent transcriptional regulation; epigenetic modification	Promote multiple aspects of tumor growth	Multiple cancer types	[17–19]
lncRNA H19	Up-regulated	HIF-1α dependent transcriptional regulation affected by SP1 or PTEN	Promote proliferation, migration, invasion, angiogenesis	Multiple cancer types	[20, 21]
lncRNA MALAT1	Up-regulated	HIF-1α dependent transcriptional regulation, HIF-1α dependent histone demethylation, chromatin looping	Promote cellular proliferation rates, EMT, and metastasis	Multiple cancer types	[22–24]
lncRNA NEAT1	Up-regulated	HIF-1α/HIF-2α dependent transcriptional regulation	Induce paraspeckle formation and accelerate cell proliferation	Multiple cancer types	[25–27]
lncRNA HOTAIR	Up-regulated	HIF-1α dependent transcriptional regulation affected by coactivator CBP/p300 or MLL1	Enhance cancer cell proliferation, migration, and invasion	Multiple cancer types	[28–30]
circRNA DENND4C	Up-regulated	HIF-1α dependent transcriptional regulation	Promote proliferation, migration, and glycolysis	Breast cancer, colorectal cancer	[31–33]
circRNA WSB1	Up-regulated	HIF-1α dependent transcriptional regulation	Promote proliferation	Breast cancer	[34]
lncRNA HAS2-AS1	Up-regulated	HIF-1α/NF-κB dependent transcriptional regulation	Promote EMT and invasiveness	Oral squamous cell carcinoma	[35]
lincRNA-p21	Up-regulated	HIF-1α dependent transcriptional regulation	Modulate the Warburg effect	Cervical cancer	[36]
circRNA MAT2B	Up-regulated	HIF-1α dependent transcriptional regulation	Facilitate glycolysis and proliferation	Gastric cancer	[37]
lncRNA PVT1	Up-regulated	HIF-1α dependent transcriptional regulation	Promote proliferation, migration, and invasion	Pancreatic cancer	[38]
circRNA ZNF91	Up-regulated	HIF-1α dependent transcriptional regulation	Enhance glycolysis and chemoresistance	Pancreatic cancer	[39]
lncRNA BCRT1	Up-regulated	HIF-1α dependent transcriptional regulation	Promote proliferation and metastasis	Breast cancer	[40]
lncRNA RAB11B-AS1	Up-regulated	HIF-2α dependent transcriptional regulation	Promote angiogenesis and metastasis	Breast cancer	[41]
lncRNA SARCC	Down-regulated	HIF-2α dependent transcriptional regulation	Suppress cell proliferation	Renal cell carcinoma	[42]
lncRNA-LET	Down-regulated	HIF-1α dependent histone deacetylation	Promote metastasis	Hepatocellular carcinomas, colorectal cancer, squamous-cell lung carcinoma	[43]
lncRNA CF129	Down-regulated	HIF-1α dependent histone deacetylation	Inhibit proliferation and invasion	Pancreatic cancer	[44]
lncRNA WT1-AS	Up-regulated	HIF-1α dependent DNA demethylation	Regulate developmental gene	Myeloid Leukemia	[45]
miRNA-424	Up-regulated	PU.1 dependent transcriptional regulation	Promote angiogenesis	Ovarian cancer	[46]
lncRNA LASTR	Up-regulated	c-JUN dependent transcriptional regulation	Foster cancer cell fitness	Triple-negative breast cancer	[47]
miRNA-148a	Down-regulated	hypermethylation of CpG islands	Promote metastasis	Multiple cancer types	[48]
miRNA-34b/c	Down-regulated	hypermethylation of CpG islands	Promote metastasis	Multiple cancer types	[48, 49]

### HIFs-dependent manner

NcRNAs can be either directly or indirectly regulated by HIFs during hypoxic stress. To be specific, ncRNAs can be directly transcribed by HIFs, on the other hand, ncRNAs can act as downstream effectors that are indirectly influenced by HIFs via other intermediate mediators. These will be further explained in the following part.

#### Direct regulation by HIFs

Hypoxia has been well-established to modulate the expression of a myriad of ncRNAs, while the majority of them are direct transcriptional targets of HIFs [16]. The HIF family is a set of heterodimeric transcription factors composed of HIF-1α, HIF-2α, or HIF-3α and a constitutively expressed HIF-1β subunit. Upon hypoxia, oxygen depletion blocks the hydroxylation and subsequent degradation of HIF-1α, thus allowing HIF-1α to translocate into the nucleus where it dimerizes with HIF-1β. This complex then binds to hypoxia response elements (HREs) in the promoter regions of target genes to regulate ncRNA transcription [4, 50]. To date, hypoxia-responsive ncRNAs that are transcribed by HIF-1α have been largely identified. These ncRNAs are validated to contain HREs in their promoter regions by bioinformatics analysis or experiments such as chromatin immunoprecipitation (ChIP), luciferase reporter assays, or electrophoretic mobility shift assay (EMSA). And these ncRNAs participate in cancer cell proliferation, metabolic reprogramming, apoptosis, metastasis, and therapy resistance in multiple cancer types [10]. Of the substantial miRNAs that are directly regulated by HIF-1α during oxygen deprivation, miR-210 is one of the most well-known and robustly upregulated miRNAs. MiR-210 overexpression has been detected in a variety of solid tumors, and a wide spectrum of its target genes have been identified, which are implicated in many stages of tumorigenesis [17]. Recently, a wealth of evidence has unraveled various lncRNAs and circRNAs that are transcriptionally regulated by hypoxia/HIF-1α signaling. For instance, lncRNA H19, MALAT1, NEAT1, and HOTAIR are frequently dysregulated upon hypoxic stress and play pleiotropic roles in many solid tumors [20–22, 25, 26, 28, 29]. Besides, the induction of circDENND4C and circWSB1 upon hypoxia are also attributed to the binding of HIF-1α to their promoters. Enhanced expression of circDENND4C facilitates glycolysis, migration, and invasion by sponging miR-200b and miR-200c or through the miR-760/GLUT1 axis in breast cancer or in colorectal cancer cells, respectively [31–33]. Overexpressed circWSB1 promotes breast cancer progression via destabilizing p53 by interacting with USP10 [34]. In addition, it is worth noting that a majority of these hypoxia-responsive ncRNAs are discovered via high-throughput RNA-seq or RNA-microarray. As an example, Zhu et al. performed a lncRNA microarray (GEO accession number: GSE84807) to analyze the aberrant lncRNA expression profiles in oral squamous cell carcinoma (OSCC) cell lines cultured under normoxia and hypoxia, screening top 10 lncRNAs that were significantly upregulated (Additional file 1: Figure S1a), among which lncRNA HAS2-AS1 was the only lncRNA directly targeted by HIF-1α and was associated with lymph node metastasis in patients with OSCC, thus being selected for further investigation. Functionally, HAS2-AS1 mediated the hypoxia-regulated EMT process via increasing HAS2 and HA production, conferring a migratory and invasive phenotype to OSCC cells [35]. Most importantly, many of these hypoxia/HIF-1α-regulated ncRNAs can conversely modulate HIF-1α expression, forming a positive/negative feedback loop as a response to hypoxic stress [26]. One such example is lincRNA-p21, which is transcriptionally induced by HIF-1α under hypoxia, can in turn interrupt the HIF-1α-VHL interaction, thereafter stabilizing HIF-1α and upregulating genes involved in glycolysis in colorectal cancer cells [36]. Likewise, in the pathogenesis of gastric cancer, the circMAT2B/miR-515-5p/HIF-1α feedback circuit reinforces HIF-1α signaling, which then amplifies the oncogenic effect of circMAT2B, eventually facilitating glycolysis [37]. Similar feedback loops also exist in the hypoxic regulation of MALAT1 [22], HOTAIR [30], lncRNA PVT1 [38], and circZNF91 [39]. Intriguingly, instead of having an impact on neoplastic cells, certain hypoxia-sensitive ncRNAs can also orchestrate the cellular behavior of various stromal cells within TME, therefore contributing to tumor progression. One such example is lncRNA BCRT1, whose expression is strongly induced in breast cancer cells as a response to hypoxia through HIF-1α-dependent transcriptional regulation. BCRT1 can be transferred from breast cancer cells to macrophages via exosomes, which promotes M2 phenotype polarization and enhances tumor-promoting properties of macrophages [40].

Similar to HIF-1α, HIF-2α is also capable of mediating the hypoxic induction of ncRNAs at transcriptional level by forming a transcription complex with HIF-1β. Importantly, it is reported that HIF-1α serves as central transcriptional mediators in acute hypoxia, while HIF-2α mainly functions in chronic hypoxia [51]. HIF-3α inhibits HIF-dependent regulation of target genes via competition with HIF-1α and HIF-2α [52]. Of note, HIF-1α and HIF-2α orchestrate the expression of both overlapping and unique sets of ncRNAs. However, compared to HIF-1α, ncRNAs that are regulated by HIF-2α remain less identified. In low oxygen tension, lncRNA RAB11B-AS1 induction is only specifically driven by HIF-2α. Silencing of HIF-2α, instead of HIF-1α, markedly attenuates the hypoxic elevation of RAB11B-AS1 expression in breast cancer cells. RAB11B-AS1 then promotes hypoxia-mediated angiogenesis and metastasis in breast cancer [41]. In contrast, miR-210, lncRNA NEAT1, and MALAT1 are induced at transcriptional level under hypoxic conditions in both HIF-1α/HIF-2α-dependent fashion. Although both HIF isoforms bind to the NEAT1 and MALAT1 locus, these two lncRNAs are regulated predominantly by HIF-2α rather than HIF-1α, indicating a post-binding mechanism of transcriptional selectivity [26, 53, 54]. Aside from transcriptional activation, it is reported that HIF-2α can transcriptionally repress lncRNA SARCC expression in renal cell carcinoma (RCC) through binding to HREs located within SARCC promoter. LncRNA SARCC functions as a key modulator that connects hypoxia/HIF-2α signaling to the AR/HIF-2α/C-MYC axis, forming a negative feedback loop and hence suppressing RCC cell proliferation [42]. Taken together, we conclude that hypoxia-responsive ncRNAs are directly regulated by HIF-1α and/or HIF-2α.

It is worth mentioning that the interactions between HIF-1α and other transcription factors or coactivators also influence ncRNA expression upon hypoxia. As an example, it is reported that lncRNA H19 elevation upon hypoxic stress is governed by HIF-1α and p53 status. Under low oxygen concentration, H19 is dramatically upregulated by HIF-1α in p53 null hepatocellular carcinoma (HCC) cells, whereas it is not affected in wild-type p53 HCC cells, suggesting that p53 interferes with the transcriptional activity of HIF-1α [55]. Alternatively, in glioblastoma (GBM), specific protein 1 (SP1) plays a crucial intermediary role in HIF-1α-mediated H19 expression via binding GC-boxes in the H19 promoter. Likewise, phosphatase and tensin homolog (PTEN) is shown to attenuate the stability of HIF-1α thereby repressing hypoxia-driven transcriptional induction of H19 in multiple GBM cell lines [20]. Similarly, transcription coactivators MLL1 and CBP/p300 are recruited to the lncRNA HOTAIR promoter region, cooperating with HIF-1α to stimulate HOTAIR expression and promote tumorigenesis [29]. Collectively, these findings indicate that HIFs-dependent direct transcriptional regulation of ncRNAs in response to hypoxia is affected by the involvement of other critical factors, however, the exact mechanisms of their actions remain to be clarified.

#### Indirect regulation by HIFs

Besides direct transcriptional regulation, HIFs have also been reported to modulate the expression of ncRNAs via indirect manners involving the participation of other intermediate factors. Mounting evidence has illustrated that hypoxia-induced HIFs influence the expression or activity of many epigenetic regulators to indirectly orchestrate epigenetic chromatin modification and subsequent gene expression [16]. For instance, it has been shown that hypoxia can alter the histone deacetylases (HDACs) levels in a HIFs-dependent manner [16]. Current studies have uncovered the role of the HIF-1α/HDACs complex in ncRNA regulation. Yang and colleagues demonstrated that lncRNA-LET downregulation in response to hypoxia was mediated by HIF-1α-induced expression of HDAC3, a downstream effector of HIF-1α. Elevation of HDAC3 blocked lncRNA-LET transcription via decreasing the level of histone H3 and H4 acetylation-mediated modulation in the lncRNA-LET promoter. Downregulation of lncRNA-LET further contributed to hypoxia-induced HCC metastasis through stabilization of NF90 protein. And the HIF-1α/HDAC3/lncRNA-LET/NF90 axis could establish a positive feedback circuit that augmented the HIF-1α response under hypoxic circumstances [43]. Similarly, HIF-1α halts the lncRNA CF129 transcription by recruiting HDAC1 to the promoter area of lncRNA CF129 under low oxygen tension. Downregulated CF129 potentiates the proliferation and invasion of pancreatic cancer cells by overexpressing FOXC2 in a p53-dependent manner. As a transcription factor, FOXC2 can reciprocally upregulate HIF-1α at transcription level, forming a lncRNA-involved regulatory feedback loop that mediates tumor progression during hypoxic stress [44]. Particularly, several HDACs were also reported to reciprocally enhance the stability and accumulation of HIFs, thus further accelerating the modulation of ncRNAs [16].

Apart from HDACs, several histone lysine demethylases (KDMs) are also known to be induced by hypoxia in a HIF-1α-dependent manner, which then regulate the expression of ncRNAs. For example, Ikeda et al. observed a HIF-1α-dependent induction of KDM3A under chronic hypoxia in multiple myeloma cell lines. KDM3A subsequently activates lncRNA MALAT1 transcription via histone demethylation at its promoter region. The HIF-1α-KDM3A-MALAT1 axis contributes to acquisition of the antiapoptotic phenotype via upregulation of glycolysis-promoting genes [23].

In addition, the study of McCarty et al. revealed another model for hypoxia-mediated epigenetic regulation of ncRNA expression: hypoxia elicits both a decrease in DNA methyltransferase 1 (DNMT1) expression and activity, while an increase in ten-eleven-translocation 2 (TET2) and ten-eleven-translocation 3 (TET3) expression and activity, resulting in demethylation of a CpG island in Intron 1 of WT1 gene, which allows the upregulation of lncRNA WT1-AS that correlates with increased mRNA WT1 expression. Notably, the hypoxic induction of WT1-AS could be abolished by HIF-1α knockdown, suggesting the critical involvement of HIF-1α [45]. Overall, these examples indicate that HIFs can indirectly regulate ncRNA expression via various forms of epigenetic modulation.

### HIFs-independent manner

Although it is well-appreciated that HIFs act as a central hub in coordinating hypoxia-responsive ncRNA expression under exposure to low oxygen levels, several studies demonstrated that silencing HIF-1α and/or HIF-2α do not block or only partially block hypoxic induction of several ncRNAs [24, 56, 57], indicating the existence of HIFs-independent regulatory pathways of ncRNAs under hypoxia. These HIFs-independent mechanisms mainly comprise transcriptional and post-transcriptional regulation.

#### Transcriptional regulation

Increasing evidence implicates that hypoxia not just stimulates HIFs expression, but also a range of other transcription factors. Transcription factors including p53, NF-κB, CREB, SP-1, ATF4, STAT3, c-Myc, c-JUN, etc., were shown sensitive to oxygen levels and drive transcriptional response to varying degrees under hypoxia [10, 58, 59], suggesting that these transcription factors may also contribute to the transcription of ncRNAs under oxygen deprivation. Many studies have shown that hypoxia can regulate miRNA expression via various kinds of transcription factors. For example, the well-studied miR-210 contains multiple conserved transcription factor recognition sites in its genomic region, including p53, NF-κB, Oct-4, and PPARγ which are hypoxia-regulated genes [18, 19]. Besides, miR-424 is upregulated in hypoxic endothelial cells by transcription factor PU.1-dependent transactivation, thus promoting angiogenesis in ovarian cancer [46]. Zhu et al. reported that hypoxia facilitated the transcription of lncRNA HAS2-AS1 via transcription factor NF-κB. HAS2-AS1 mediated the hypoxia-induced invasive properties of OSCC cells via stabilizing HAS2 [35]. Another recent study validates that the expression of lncRNA LASTR is triggered by stress-activated JNK/c-JUN pathway under hypoxia through binding of the transcription factor c-JUN to LASTR transcription start site. In silico prediction and ENCODE ChIP-seq did not reveal any HREs located in the LASTR promoter. In this study, LASTR is selected as a representative lncRNA following RNA-seq (GEO accession number: GSE129344) and TCGA data analysis, showing robustly upregulated expression upon hypoxia (Additional file 1: Figure S1b) and close relevance to breast cancer malignancy. LASTR overexpression fosters cancer cell fitness by regulating the activity of the U4/U6 recycling factor SART3 [47]. These results confirmed that the transcription of ncRNAs can be modulated by other transcription factors other than HIFs in low oxygen tensions. Nevertheless, more transcription factors apart from c-JUN require to be identified.

Chromatin reprogramming, which is a well-established phenomenon during pathological conditions like cancer, also frequently emerges in hypoxic TME and regulates the transcription of a broad spectrum of genes in response to hypoxia [60, 61]. As an example, the hypoxia-induced hypermethylation of CpG islands leads to the transcriptional silencing of miR-148a and miR-34b/c, which promotes the metastasic capabilities of various cancers [48, 49]. Additionally, a recent research uncovers that hypoxia triggers cancer-cell-specific chromatin-chromatin interactions between enhancer-like cis-regulatory elements present at the lncRNA MALAT1 locus, which facilitates transcription of MALAT1 in breast cancer. Functionally, MALAT1 overexpression elevates the proliferation rate, EMT process, and metastatic potential of breast cancer cells. In this study, researchers indicate that hypoxia can stimulate MALAT1 expression in both HIFs-dependent and HIFs-independent manners, and they predict that numerous chromatin looping events remain to be uncovered which might influence hypoxia-responsive ncRNA expression [24].

#### Post-transcriptional regulation

NcRNA biogenesis is a complicated multistep process that integrates various enzymes and regulatory proteins to ensure an accurate maturation of ncRNAs, whereas, in multiple human malignancies, many components of the ncRNA biogenesis machinery could be deregulated, leading to aberrant ncRNA expression. For instance, it is well-established that miRNA processing undergoes dysregulation upon hypoxic stress, as a result of the significant downregulation of key proteins like DROSHA and DICER and many other miRNA processing subunits [62]. Besides, as one of the post-transcriptional gene regulatory mechanisms, alternative splicing (AS) has been proven to have an impact on the biogenesis and biological behavior of miRNAs, lncRNAs, and circRNAs [63, 64]. Importantly, it has been demonstrated that hypoxia could trigger abnormal AS events in cancer [58]. Nevertheless, the AS dysregulation of lncRNAs and circRNAs in low oxygen availability remains greatly unexplored with regard to their molecular mechanisms and biological functions in tumor pathogenesis. In addition, hypoxia can activate adenosine-to-inosine RNA sequence editing in cancer cells, which is also a post-transcriptional means of noncoding gene regulation [65, 66]. Furthermore, it was previously reported that under low oxygen tension, RNA-binding proteins (RBPs) and miRNAs could bind to mRNAs and impact mRNA turnover to modulate hypoxic gene expression profile [67]. Similarly, ncRNAs (e.g., lncRNAs, circRNAs) are recently shown to physically bind with RBPs and miRNAs, which can affect ncRNA biogenesis, stability, and function [68–70]. However, it remains unknown whether this post-transcriptional modification of ncRNAs can also be affected by hypoxia.

In conclusion, these studies provide further knowledge on the diverse mechanisms of hypoxia-triggered alterations of ncRNA expression, adding another layer of complexity in understanding how a hypoxic TME is implicated in cancer formation and progression.

## NcRNAs regulated by inflammation

**Fig. 2:**
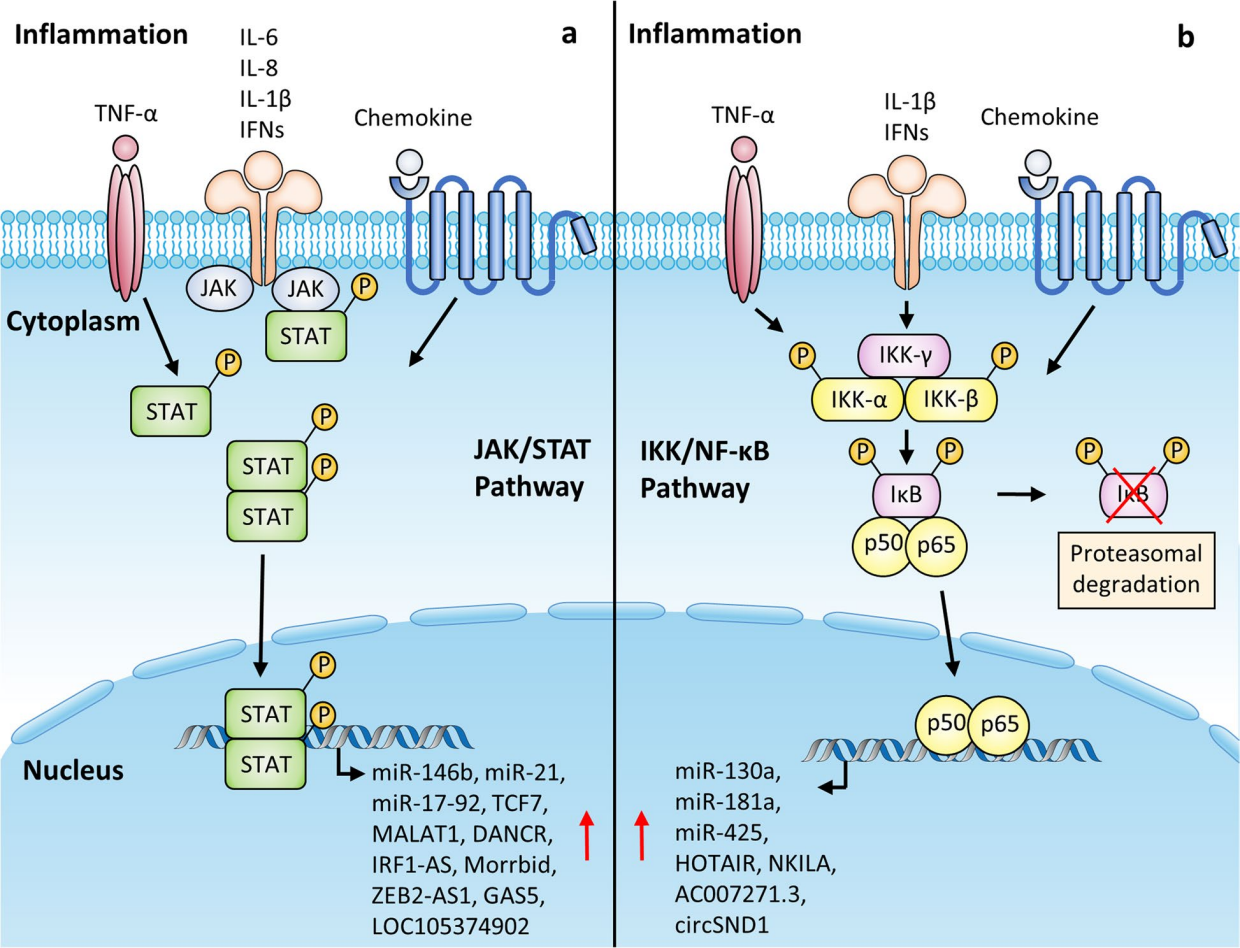
Schematic illustration of JAK/STAT and IKK/NF-κB pathway linking inflammation and ncRNA transcription in TME. a Various inflammation-associated cytokines (IL-6/IL-8/IL-1β/TNF-α/IFNs) and chemokines bind directly to their receptors on cancer cell membrane and activate JAK tyrosine kinase. Activated JAK tyrosine kinase phosphorylates STAT. Phosphorylated STAT dimerizes and translocates into the nucleus, where it binds to the gene promoter regions and transcribes numerous ncRNAs. b In response to pro-inflammatory cytokines like TNF-α, IL-1β, IFNs, or chemokines, the formation of IKK protein complex, which consists of IKK-α, β, γ subunits, is activated. The IKK complex then phosphorylates the IκB subunit of NF-κB/IκB complex, thereby resulting in proteasomal degradation of IκB and the nuclear translocation of NF-κB dimers (p50/p65). The NF-κB (p50/p65) complex mediates the transcription of various noncoding genes through binding to their promoter regions

Chronic inflammation, as one of the defining hallmarks of cancer, is commonly observed in multiple human malignancies [71]. Considerable progress has been made in understanding the association between cellular/molecular events under inflammatory stimuli and tumor development. Under chronic inflammation in TME, stromal cells, infiltrating immune cells, and neoplastic cells produce a myriad of inflammatory factors that orchestrate an intricate inflammatory signaling network to modulate tumorigenesis and progression. These inflammatory factors include a variety of cytokines, chemokines, and growth factors [2, 72]. A growing body of evidence has revealed that inflammatory factors exert regulatory effects on ncRNAs via various pathways, such as Janus kinase-signal transducer and activator of transcription (JAK/STAT) signaling pathway, nuclear factor-κB (NF-κB) signaling pathway, or chromatin remodeling, etc. Intriguingly, emerging evidence has indicated that the regulation of ncRNAs by inflammation in TME is not a one-way road. Many ncRNAs which are under regulation by inflammatory stimuli, can contribute to the production of a variety of inflammatory factors, which may in turn aggravate or repress the cancer-related inflammation. In the following context, we present an overview of the distinct mechanisms through which ncRNAs are induced by various inflammatory factors while highlighting how these inflammation-associated ncRNAs play pivotal roles in linking inflammation to malignant phenotype (Table 2, Fig. 2).

**Table 2:** Inflammation-regulated ncRNAs and their roles during cancer progression

NcRNAs	Expression in inflammation	Regulatory mechanisms	Functions	Cancer types	References
miRNA-21	Up-regulated	IL-6/STAT3, IFN/JAK/STAT3 or IFN/NF-κB	Promote metastasis, modulate immune response	Multiple cancer types	[73, 74]
miRNA-17–92	Up-regulated	IL-6/STAT3	Enhance proliferation, and invasiveness	Cholangiocarcinoma	[75]
miRNA-146b	Up-regulated	IL-6/STAT3	Inhibit migration and invasion	Breast cancer	[76]
lncRNA TCF7	Up-regulated	IL-6/STAT3	Promote EMT	Hepatocellular carcinoma	[77]
lncRNA DANCR	Up-regulated	IL-6/STAT3	Promote sorafenib resistance	Hepatocellular carcinoma	[78]
lncRNA Morrbid	Up-regulated	IL-6/STAT3	Inhibit apoptosis	Acute myeloid leukemia	[79]
lncRNA ZEB2-AS1	Up-regulated	IL-6/STAT1	Enhance migration	Non-small cell lung cancer	[80]
circRNA cGGNBP2	Up-regulated	Circularization inhibition via DHX9	Promote cell growth and metastasis	Intrahepatic cholangiocarcinoma	[81]
lncRNA MALAT1	Up-regulated	IL-6/STAT3, IL-8/STAT3 or CCL21	Promote cell proliferation and metastasis	Multiple cancer types	[82–85]
miRNA-181a	Up-regulated	IL-1β/NF-κB	Promote cell proliferation	Colorectal cancer	[86]
miRNA-425	Up-regulated	IL-1β/NF-κB	Promote cell proliferation	Gastric cancer	[87]
lncRNA NKILA	Up-regulated	TNF-α/NF-κB	Suppress proliferation, metastasis	Breast cancer; laryngeal cancer	[88, 89]
circRNA SND1	Up-regulated	TNF-α/NF-κB	Promote migration and invasion	Cervical cancer	[90]
miRNA-130a	Up-regulated	TNF-α/NF-κB	Promote cell growth	Cervical cancer	[91]
lncRNA AC007271.3	Up-regulated	TNF-α/NF-κB	Promote proliferation, migration, invasion, and inhibit apoptosis	Oral squamous cell carcinoma	[92]
lncRNA HOTAIR	Up-regulated	TNF-α/NF-κB or CCL18	Promote proliferation, angiogenesis, drug resistance	Multiple cancer types	[93, 94]
lncRNA LOC105374902	Up-regulated	TNF-α/STAT3	Facilitate EMT process, migration, invasion	Cervical cancer	[95]
lncRNA IRF1-AS	Up-regulated	IFN/JAK/STAT	Inhibit proliferation and promote apoptosis	Esophageal squamous cell carcinoma	[96]
lncRNA GAS5	Up-regulated	IFN/JAK/STAT	Inhibit proliferation, migration, and invasion	Esophageal squamous cell carcinoma	[97]
lncRNA LINC00092	Up-regulated	CXCL14	Promote glycolysis and metastasis	Ovarian cancer	[98]
lncRNA XIST	Up-regulated	CXCL12/CXCR4	Promote invasion and metastasis	Colorectal cancer	[99]
lncRNA LINC00319	Up-regulated	CCL18	Promote proliferation and metastasis	Oral squamous cell carcinoma	[100]
circRNA circ_0026344	Down-regulated	CCL20 and CXCL8 co-stimulation	Restrain metastasis	Colorectal cancer	[101]

### Cytokines

In inflammatory TME, cytokines like interleukins (ILs), tumor necrosis factors (TNFs), and interferons (IFNs) can be either autocrine, produced by neoplastic cells, or paracrine, released from adjacent stromal cells or immune cells [71]. These cytokines are demonstrated to drive aberrant expression of ncRNAs, which exert diverse functions in cellular processes during cancer initiation and malignant progression.

#### Interleukins

A variety of interleukins are abnormally produced in the presence of inflammatory stimuli. As a consequence, many inflammatory signaling pathways such as IL-6/JAK/STAT3 axis are frequently hyperactivated in multiple human cancers, executing pro-tumorigenic functions. The elevated levels of phosphorylated STAT3 are observed to translocate into the nucleus and initiate transcription of a vast array of ncRNAs [102]. To date, the IL-6/STAT3 axis has been well-documented to manipulate the expression of numerous miRNAs, including miR-21 [73], miR-17–92 [75], and miR-146b [76], and establish various regulatory feedback loops which further impact malignant progression. For example, IL-6/STAT3-induced miR-17–92 targets JAK/STAT3 pathway inhibitor SOCS3 and PIAS3, generating a positive feedforward loop that amplifies IL-6/STAT3 signaling and contributes to cholangiocarcinoma cell proliferation and invasion [75]. Likewise, activation of IL-6/STAT3 axis induces lncRNA TCF7 expression in HCC cells. The binding of STAT3 to the promoter region of lncTCF7 upon IL-6 stimulation is confirmed by luciferase reporter assay and ChIP assay. In HCC, the aberrant IL-6/STAT3/lncTCF7 signaling axis boosts a malignant phenotype by accelerating the EMT process of HCC cells [77]. Besides, the IL-6/STAT3 triggers transcription of lncRNA DANCR. Overexpression of DANCR then stabilizes PSMD10 which in reciprocal promotes IL-6 production, thus forming a feedback regulatory loop in reinforcing a pro-inflammatory TME and enhancing sorafenib resistance of HCC cells [78]. Another study reveals that under inflammatory stress, the hyperactivated IL-6/STAT3 axis in preleukemic Tet2-deficient hematopoietic stem and progenitor cells (HSPCs) results in elevated expression of lncRNA Morrbid via increased binding of STAT3 to the Morrbid promoter, which impedes apoptosis and enhances survival of Tet-KO HSPCs in response to acute inflammatory insults, ultimately promoting myeloid malignancies [79]. Similar to the IL-6/STAT3 pathway, IL-6 was shown to upregulate the expression of lncRNA ZEB2-AS1 through STAT1 pathway, which fostered the proliferation and migration of non-small cell lung cancer cells [80].

Besides, a recent study unravels that IL-6 could interfere with circRNA biogenesis. DEx-H Box Helicase 9 (DHX9) is an RBP which is verified to disrupt the circularization of circRNA cGGNBP2 by binding to its flanking inverted complementary sequences in intrahepatic cholangiocarcinoma (ICC). However, upon IL-6 treatment, DHX9 is markedly downregulated, thus attenuating its inhibitory effects on the biogenesis of cGGNBP2. The upregulated cGGNBP2 thereby promotes ICC cell proliferation and metastasis in vitro and in vivo by encoding a novel protein named cGGNBP2-184aa. Notably, cGGNBP2 also participates in a positive feedback loop involving IL-6, cGGNBP2-184aa, and STAT3, which sustains the constitutive activation of inflammatory IL-6/STAT3 signaling and accelerates ICC progression [81].

In addition to IL-6, Zheng et al. demonstrated that IL-8 secreted by M2 macrophages triggered the transcriptional activation of MALAT1 via STAT3 pathway in inflamed prostate cancer cells, while upregulated MALAT1 expression then promoted cellular proliferation and metastasis [82]. The inflammatory cytokine IL-1β has been found to modulate the expression of various miRNAs linking inflammation to cancer progression. For instance, the elevated expression of miR-181a in colon cancer and miR-425 in gastric cancer are both attributed to activated IL-1β/NF-κB signaling axis, and they are both able to promote cancer cell proliferation by repressing tumor suppressor PTEN, which is frequently mutated in many human malignancies [86, 87]. However, further investigations are warranted to determine whether other interleukins (e.g., IL-2, IL-10, IL-11, IL-12, IL-17, IL-23) can orchestrate ncRNA expression.

#### Tumor necrosis factors

As a kind of multifunctional pro-inflammatory cytokine in TME, TNFs are capable of activating multiple inflammatory signaling cascades which consequently drive differential protein-coding and noncoding transcriptome profiles [103]. As an example, Liu and colleagues identified a lncRNA NKILA which showed dramatic upregulation upon TNF-α treatment in breast cancer cells and suppressed breast cancer metastasis. Initially, the authors conducted RNA-microarray (GEO accession number: GSE57539) to screen differentially expressed lncRNAs in breast cancer cells upon treatment with various inflammatory mediators, and identified NKILA as a representative due to its significant stimulation upon all kinds of inflammatory stimuli (Additional file 1: Figure S1c). Further mechanistic study demonstrated that TNF-α upregulated NKILA at transcriptional level via NF-κB signaling, specifically with the NF-κB complex binding to the promoter region of NKILA. NKILA served as a tumor suppressor which in turn bound to NF-κB/IκB complex and repressed NF-κB signaling, forming a negative feedback loop that finally blocked the over-activation of NF-κB signaling in inflammation-stimulated breast epithelial cells [88]. Notably, a similar negative feedback loop was also observed in laryngeal cancer which sensitized cancer cells to X-ray radiation [89]. Besides, TNF-α was recently reported to induce the expression of circSND1 via activating NF-κB pathway. CircSND1 promoted the EMT process and metastatic potential of cervical cancer cells. Of note, circSND1 elevation could in turn activate NF-κB pathway through miR-125a-3p/FUT6 axis which promoted the nucleus accumulation of p65, hence establishing a positive regulatory circuit that reciprocally stimulated inflammation and the malignant progression of cervical cancer [90]. Similarly, the increased expression of miR-130a in cervical cancer [91], upregulated lncRNA AC007271.3 in oral squamous cell carcinoma [92], and induced lncRNA HOTAIR expression in several human tumor entities [93] are also attributed to the activated TNF-α/NF-κB signaling cascade under inflammatory stimuli. Alternatively, studies have observed the TNF-α/STAT3 axis in the upregulation of various lncRNAs like LOC105374902. TNF-α is also found to potentiate the transcriptional activity of STAT3. Overexpressed LOC105374902 acts as a competing endogenous RNAs that competitively sponges miR-1285-3p to derepress RPL14, therefore facilitating the EMT process, migratory and invasive capacity of cervical cancer cells [95].

#### Interferons

IFNs are pleiotropic cytokines that exert vital roles in activating immune responses in malignant cancer [104]. Existing literature has documented that Type I IFN induces miR-21 expression via both JAK/STAT3 and NF-κB signaling pathways [74]. Huang et al. comprehensively analyze IFN-regulated lncRNAs via performing RNA-seq of IFN-β-treated and control esophageal squamous cell carcinoma (ESCC) cells (GEO accession number: GSE124514), and identifies a novel IFN-inducible nuclear lncRNA IRF1-AS in ESCC (Additional file 1: Figure S1d). IFNs elevate IRF1-AS expression via the classical JAK/STAT signaling pathway. Functionally, IRF1-AS functions as a tumor suppressor which restrains ESCC proliferation and induces apoptosis in vitro and in vivo. Meanwhile, an in-depth mechanistic study reveals that IRF1-AS enhances the transcriptional activation of IRF1 through interacting with ILF3 and DHX9. As an important transcription factor, IRF1 then conversely binds to the IRF1-AS promoter and activates IRF1-AS transcriptional activity, while also elevating the production of IFNs and transcription of many IFN-stimulated genes, ultimately forming a positive regulatory loop that stimulates the IFN response and IFN pathway gene expression to elicit a tumor-suppressive role in ESCC [96]. Meanwhile, Huang et al. also identify another IFNs-inducible lncRNA GAS5 in ESCC which shows similar modes of action as lncRNA IRF1-AS. GAS5 interacts with the IFN signaling and also forms a positive regulatory circuit that displays antitumor effects [97].

### Chemokines

Chemokines are small, secreted proteins that are well-known for their roles in mediating immune cell trafficking and stimulating cancer growth and metastasis [105]. CC-chemokine ligand 21 (CCL21), which was remarkably upregulated in both gastric cancer and leukemia, induces the overexpression of oncogenic lncRNA MALAT1 which activates the mTOR signaling pathway, thereafter expediting the EMT process to enhance the migratory and invasive capabilities of gastric cancer cells and cutaneous lymphoma cells [83, 84]. In addition, CXC-chemokine ligand 14 (CXCL14), which is secreted by CAFs, stimulates the expression of LINC00092 in ovarian cancer cells. According to lncRNA microarray analysis (GEO accession number: GSE82059), LINC00092 is among the top five most upregulated lncRNAs treated with recombinant CXCL14 protein in ovarian cancer cells (Additional file 1: Figure S1e). As for the functional mechanism, LINC00092 binds to the fructose-2,6-biphosphatase PFKFB2, a glycolytic enzyme, to alter glycolysis of ovarian cancer cells and maintain the CAFs-like phenotype of fibroblasts in TME, subsequently accelerating metastasis. This study highlights a reciprocal feedback loop mediated by LINC00092 between CXCL14-positive CAFs and ovarian cancer cells that is crucial for promoting the metastatic behavior of ovarian cancer [98]. Additionally, in the inflammation-driven colorectal cancer (CRC) cells, the activated CXCL12/CXCR4 axis results in a robust increase in lncRNA XIST, which attenuates repression of RhoA signaling by sponging miR-133a-3p, thus enhancing the cytoskeletal reorganization and invasive capacities of CRC cells [99]. Moreover, CCL18 is shown to induce the expression of lncRNA HOTAIR in ESCC. HOTAIR functions as a miR-130a-5p sponge to positively regulate ZEB1, leading to the invasiveness of ESCC cancer cells [94]. Meanwhile, CCL18 also enhances the proliferation and metastatic potential of oral squamous cell carcinoma cancer cells via LINC00319/miR-199a-5p/FZD4 signaling pathway [100]. Aside from lncRNAs, circ_0026344 is downregulated by CCL20 and CXCL8 synergized treatment in CRC cell lines. Circ_0026344 knockdown alleviates the inhibitory effects on CRC cell migration and invasion through restoring miR-183-dependent Wnt/β-catenin signaling pathway [101]. To sum up, similar to cytokines, chemokines, which were previously illustrated to modulate protein-coding gene expression via STAT or NF-κB pathway [103, 106], are also able to regulate the expression of numerous ncRNAs. However, the precise molecular mechanism regarding ncRNA regulation by chemokines was not clarified in the above studies.

In spite of the aforementioned inflammatory factors, a plethora of other inflammatory mediators also participates in tumor-related inflammation. Nevertheless, whether and how these inflammatory factors manipulate ncRNA expression has not been thoroughly elucidated.

## NcRNAs regulated by nutrient deprivation

**Fig. 3:**
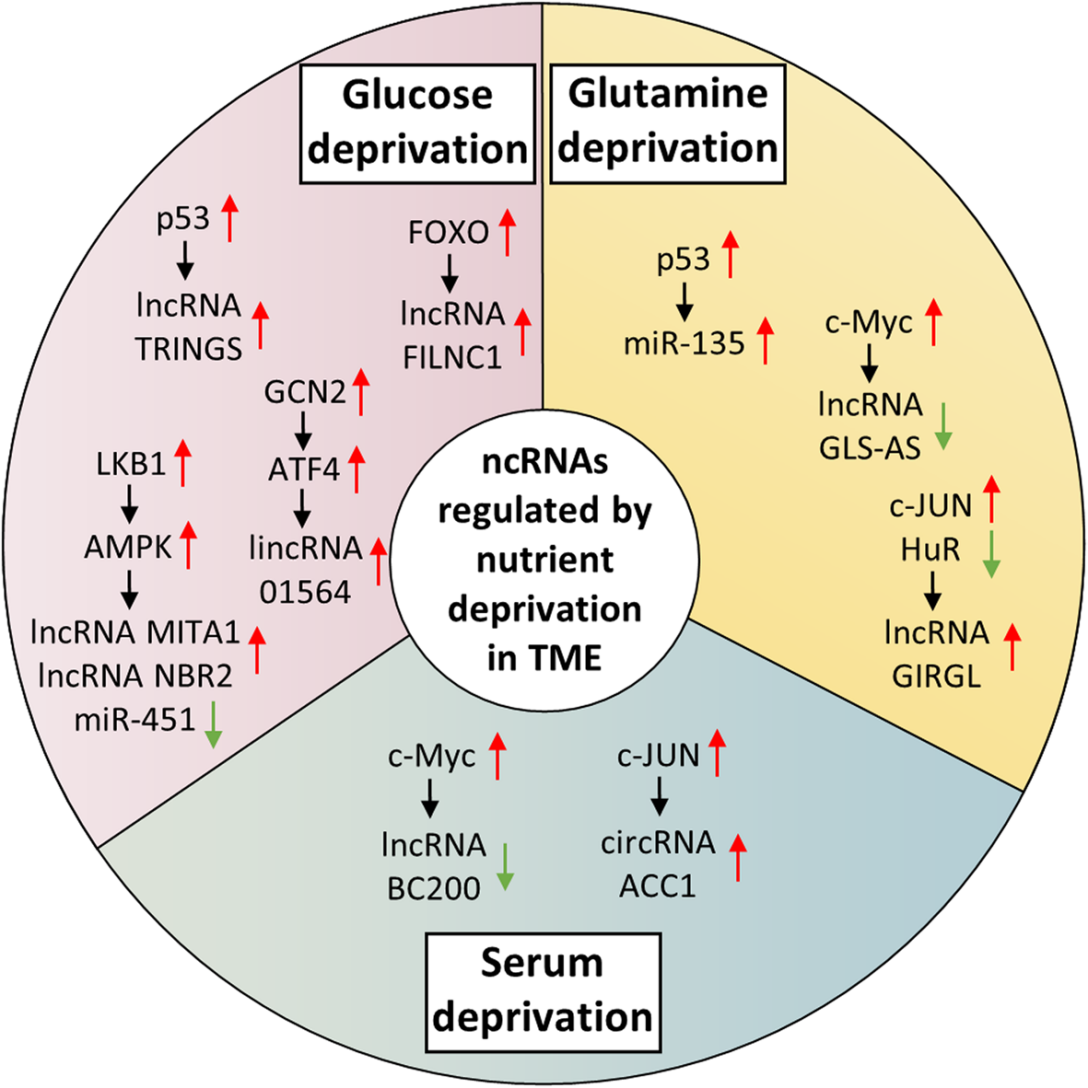
Regulatory mechanisms of nutrient deprivation on ncRNAs in TME. Nutrient deprivation, such as glucose, glutamine, and serum deprivation, modulates the expression level of various ncRNAs through multiple pathways in TME

Nutrient deprivation represents one of the main features of TME. Cancer cells generally consume a diversity of nutrients in amounts that substantially exceed those of normal cells, especially glucose and glutamine which serve as two major nutrient sources for cancer cells. To survive in the nutritionally restricted TME, various nutrient-sensing mechanisms and metabolic machineries are observed to orchestrate the metabolic reprogramming of cancer cells [107–110]. Notably, ncRNAs emerge as key players in these metabolic adaptive responses. Accumulating evidence has illustrated that an array of ncRNAs are dysregulated under nutrient-limiting conditions. These ncRNAs exert profound roles in metabolic alterations and malignant transformation of cancer cells. Previous literature primarily focuses on identifying the biological roles and functional mechanisms of ncRNAs during cancer metabolism, which have been systematically reviewed elsewhere [111–114]. However, the information about the regulatory pattern of ncRNAs has comparatively lagged behind, in contrast to the wealth of publications concerning their biological functions. Therefore, in this section, we provide current knowledge on the underlying mechanisms by which ncRNAs are regulated under different kinds of nutrient stress (Table 3, Fig. 3).

**Table 3:** Nutrient deprivation-regulated ncRNAs and their roles during cancer progression

NcRNAs	Expression upon nutrient deprivation	Regulatory mechanisms	Functions	Cancer types	References
miRNA-451	Down-regulated under glucose deprivation	AMPK/OCT1	Promote adaptive response	Glioblastoma	[115]
lncRNA MITA1	Up-regulated under glucose starvation	AMPK-mediated DNA methylation	Enhance EMT transition	Hepatocellular carcinoma	[116]
lncRNA NBR2	Up-regulated under glucose starvation	LKB1/AMPK	Regulate proliferation, apoptosis, and autophagy	Breast cancer, renal cancer	[117]
lncRNA TRINGS	Up-regulated under glucose starvation	p53	Regulate cancer cell necrosis	Multiple cancers	[118]
lincRNA 01,564	Up-regulated under glucose deprivation	GCN2/ATF4 axis	Mediate metabolism remodeling	Hepatocellular carcinoma	[119]
lncRNA FILNC1	Up-regulated under glucose deprivation	FOXO	Regulate energy metabolism	Renal cancer	[120]
lncRNA MACC1-AS1	Up-regulated under glucose deprivation	N.A	Promote cancer cell metabolic plasticity and survival	Gastric cancer	[121]
lncRNA LOC730101	Up-regulated under glucose deprivation	N.A	Regulate cancer cell survival, viability, apoptosis	Osteosarcoma	[122]
lncRNA GLCC1	Up-regulated under glucose starvation	N.A	Enhance glycolysis, support cell survival, and proliferation	Colorectal cancer	[123]
lncRNA HAND2-AS1	Up-regulated under glucose deprivation	N.A	Repress glycolysis	Osteosarcoma	[124]
lncRNA HOXC-AS3	Up-regulated under glucose deprivation	N.A	Trigger metabolic reprogramming	Breast cancer	[125]
miRNA-135	Up-regulated under glutamine deprivation	p53	Suppress glycolysis and promote tumor growth	Pancreatic ductal adenocarcinoma	[126]
lncRNA GLS-AS	Down-regulated under glucose/glutamine deprivation	c-Myc	Facilitate cancer cell proliferation and invasion	Pancreatic cancer	[127]
lncRNA GIRGL	Up-regulated under glutamine deprivation	c-JUN and HuR	Support cancer cell survival	Colon cancer	[128]
lncRNA BC200	Down-regulated under serum deprivation	c-Myc	Reduce cancer cell viability	Breast cancer, lung cancer	[129]
circRNA ACC1	Up-regulated under serum deprivation	c-JUN	Support tumor growth	Colorectal cancer	[130]
circRNA Hsa_circ_0062682	Up-regulated under serum and serine deprivation	N.A	Promote serine synthesis and tumor growth	Colorectal cancer	[131]

### Glucose deprivation

Glucose, as a central bioenergetic source, is widely consumed by cancer cells at high levels to fuel their growth. Increased glucose catabolism depletes local supply and results in glucose deprivation [132, 133]. Glucose availability modulates several signaling pathways and activity of numerous transcription factors, thus altering the transcriptional program of certain sets of noncoding genes. As an example, an increase in cellular AMP/ATP ratio caused by glucose deprivation activates the adenosine monophosphate-activated protein kinase (AMPK), which serves as a principal sensor of cellular energy status [134, 135]. The AMPK signaling pathway has been demonstrated to provide a link between nutrient-deprived signals and ncRNA regulation. One of such cases in GBM is that AMPK activation upon glucose starvation results in inactivation of transcription factor OCT1 via direct phosphorylation at serine 335, which subsequently disrupts the transcription of miR-451. Inhibition of miR-451 then conversely sustains activation of AMPK signaling and a robust response to low glucose availability in TME. This study highlights a reciprocal negative feedback loop mechanism that render GBM cells adaptive to nutrient stress [115]. In addition, under conditions of glucose depletion, activated LKB1/AMPK pathway stimulates the expression of lncRNA MITA1. Mechanistically, AMPK mediates the increased binding of DNMT3B to the CpG island within the second intron of the MITA1 locus. Overexpression of MITA1 further promotes the EMT transition of HCC cells in vitro and HCC metastasis in vivo by upregulating Slug transcription [116]. Another lncRNA NBR2 is also induced under glucose-deprived conditions via the LKB1/AMPK pathway. At the beginning of this study, to identify nutrient-stress-induced lncRNAs, the authors perform an RNA-seq analysis (GEO accession number: GSE77415) to establish lncRNA expression profile in kidney cancer cells cultured in glucose-containing or glucose-free medium. Subsequent computational analysis discovers NBR2 to be markedly induced by glucose starvation (Additional file 1: Figure S1f). Induced lncRNA NBR2, in turn, interacts with AMPK and potentiates AMPK kinase activation, forming an NBR2-AMPK feedforward loop that contributes to tumor growth. NBR2 functions as a tumor suppressor in human breast and renal cancer. Depletion of NBR2 leads to unchecked cell cycling, altered apoptosis response, and enhanced tumor development [117].

Moreover, transcription factor p53 has been well-documented to be upregulated and modulate gene expression under diverse intrinsic and extrinsic stimuli including nutrient scarcity [136]. Various lncRNAs are direct transcriptional targets of activated p53 under glucose-deprived conditions. For example, Khan and colleagues observed that in the presence of low glucose availability, p53 elevation transcriptionally upregulates the lncRNA TRINGS in multiple cancer cell lines. TRINGS physically interacts with STRAP and inhibits STRAP-GSK3B-NF-κB necrotic signaling to protect cancer cells against necrosis. It is interesting to note that TRINGS only specifically responds to glucose starvation, while its expression is not affected by other nutritional deficiencies such as serum, serine, or glutamine deprivation [118].

In addition, the GCN2/ATF4 signaling axis is recently observed to mediate linc01564 induction in HCC cells under low-glucose conditions. Linc01564 increases PHGDH expression through sequestering miR-107/103a-3p, hence activating serine synthesis pathway to support HCC cell survival and proliferation in vitro and xenograft tumor growth in vivo [119]. Besides, another lncRNA FILNC1 was induced by activated transcription factor FOXO in renal cancer cells under glucose deficiency. Elevated FILNC1 sequesters AUF1 from binding c-Myc mRNA, leading to downregulation of c-Myc protein which subsequently represses energy metabolism and impedes renal tumor development [120]. Despite these above-mentioned ncRNAs, many other ncRNAs were also revealed to be upregulated by glucose deprivation, including lncRNA MACC1-AS1 [121], lncRNA LOC730101 [122], lncRNA GLCC1 [123], lncRNA HAND2-AS1 [124], and lncRNA HOXC-AS3 [125], which are implicated in cellular processes such as metabolic reprogramming, survival, apoptosis, and proliferation in many malignancies, whereas the exact molecular mechanisms underpinning their increased expression remain elusive.

### Other nutrient deprivation

Similar to glucose, glutamine is another essential nutrient in cancer metabolism. Due to surged glutamine usage and inadequacies of tumor vascular supply, aggravated glutamine deprivation occurs in the tumor core regions [110]. NcRNAs are shown to be dysregulated under low-glutamine conditions through various mechanisms. For instance, miR-135 accumulates specifically as a response to glutamine deprivation in pancreatic ductal adenocarcinoma (PDAC). Mechanistically, glutamine depletion induces ROS which activates mutp53 and potentiates the binding of mutp53 to miR-135 promoter region, thereby inducing miR-135 transactivation. Functionally, miR-135 targets PFK1, resulting in suppressed glycolysis and enhanced cancer growth [126]. Alternatively, in pancreatic cancer cells, lncRNA GLS-AS is transcriptionally inhibited by induced c-Myc upon glutamine depletion. Downregulated GLS-AS mediates the reciprocal feedback loop of glutaminase and Myc, hence supporting pancreatic cancer cell survival and dissemination during nutrient stress. Notably, results proved that GLS-AS repression is only observed during glutamine and glucose starvation [127]. Moreover, since it has been previously established that glutamine insufficiency invokes stress-activated JNK/c-JUN signaling [137, 138], a study recently characterizes a lncRNA named GIRGL which is transactivated by c-JUN in colon cancer cells deprived of glutamine. In addition, beyond transcription control, it is also observed that GIRGL turnover can be restrained via a human antigen R (HuR)/Ago2-mediated mechanism. RBP HuR, which is known for destabilizing mRNA and lncRNA transcript or modulating mRNA translation [139, 140], binds to GIRGL transcript and accelerates its degradation through recruitment of RISC. However, this negative regulation of GIRGL by HuR appears to be diminished in the absence of glutamine. Collectively, the dual mechanisms establish a feedback loop serving to balance cellular GIRGL levels in response to fluctuating glutamine concentration. Upregulation of GIRGL under glutamine-deficient conditions drives the formation of GIRGL/CAPRIN1/glutaminase-1 mRNA complex and promotes phase separation of this complex into stress granules, resulting in the translational suppression of glutaminase-1 mRNA which eventually supports cancer cell survival upon prolonged glutamine deprivation stress [128]. Despite glutamine, the levels of other amino acids, including serine, asparagine, arginine and aspartic acid are also significantly decreased in tumor core region [141], whereas much less is known with respect to whether these types of nutrient limitations affect ncRNAs or not.

Besides glucose and glutamine deprivation, a limited supply of serum components also frequently occurs in TME, which is referred to as serum deprivation [142]. A study reports that lncRNA BC200 expression is inactivated via c-Myc upon serum deprivation. Knockdown of BC200 reduces cell survival and proliferation in a spectrum of cancer cell lines [129]. Similarly, serum deprivation upregulates circACC1 in colorectal cancer cells by transcription factor c-JUN. CircACC1 contributes to the metabolic switch in response to serum deprivation. Mechanistically, circACC1 assembles and stabilizes the AMPK complex, ultimately increasing both glycolysis and fatty acid β-oxidation that further support colorectal cancer development [130]. Lastly, it is reported that Hsa_circ_0062682, a serum and serine starvation-induced circRNA, promotes serine metabolism and tumor growth via miR-940/PHGDH axis in colorectal cancer [131].

As discussed above, nutrient deprivation in TME could contribute to aberrant expression of ncRNAs in cancer cells through a series of transcriptional/post-transcriptional perturbations and epigenetic alterations. However, further studies are required to unravel other direct or indirect regulatory mechanisms. For instance, investigations are required to identify more nutrient-stress-responsive RBPs that could elicit post-transcriptional regulation on ncRNAs. Furthermore, since both glucose and amino acid depletion were reported to induce mTOR-MDM2-Drosha axis that broadly orchestrated miRNA biogenesis in lung cancer cells [143], we propose that nutrient deficiency might also affect lncRNA and circRNA biogenesis via similar pathways. Moreover, in response to low nutrient availability, a variety of activated metabolic enzymes and secreted metabolites can impact chromatin structure and transcription directly or indirectly by modulating histone-modifying enzymes, chromatin-remodeling complexes, and transcription regulators, displaying potential impact on ncRNA expression. Besides, the interplays between deregulated metabolic activities and various kinds of covalent modifications have been validated to exert regulation on gene expression in tumor development, which also implies potential regulatory effects on expression of ncRNAs [144].

## NcRNAs regulated by other tumor environmental stresses

As tumor develops, in spite of these above-described stresses, endoplasmic reticulum (ER) stress, oxidative stress, acidosis also occur prevalently in TME and display modulatory effects on ncRNA expression (Table 4). Adverse external and internal factors result in accumulation of unfolded or misfolded proteins in the endoplasmic reticulum in cancer cells, which triggers a stressful condition termed ER stress and elicit the unfolded protein response (UPR) to restore intracellular homeostasis. To adapt to unfavorable conditions, some transcription factors downstream of UPR sensors strictly coordinate transcriptional activities to ensure gene expression. Increasing evidence indicates that ER stress affects the expression level of miRNAs and lncRNAs, whereas there are barely any reports concerning circRNAs [14]. For example, an ER-stress-responsive C/EBP-homologous protein (CHOP) is a transcription factor that regulates many genes involved in ER-stress-induced apoptosis. CHOP is reported to transactivate lncRNA GOLGA2P10 by directly binding to its promoter region. Overexpression of GOLGA2P10 enables HCC cells to evade ER-stress-triggered apoptosis and survive under stress conditions by increasing BCL-xL protein level and promoting BAD phosphorylation [145]. Another report from Jiang et al. reported that upon ER stress, lncRNA MALAT1 expression was increased via activation of IRE1/XBP1 and PERK/elF2α/ATF4 signaling pathways. Augmented lncRNA MALAT1 then promotes colorectal cancer metastasis [146]. Interestingly, crosstalk between ER stress and ncRNAs has been identified during tumorigenesis and progression. Many transcription factors downstream of ER-stress-triggered UPR signaling pathway bind to the promoter regions of ncRNAs to activate their transcription, and certain ncRNAs can reciprocally modulate the UPR signaling pathway [14]. One of such example is the aforementioned lncRNA GOLGA2P10, which forms a feedback loop with the CHOP signaling to prevent tumor cells against CHOP-induced apoptosis [145]. Particularly, ER stress is reported to stimulate exosomal secretion of ncRNAs, which promotes the dynamic intercellular communications between various cell types in TME. As an example, Liu et al. reveal that ER stress contributes to exosome secretion and elevated exosomal miR-23a-3p level in HCC cells. The miR-23a-3p-enriched exosomes can be taken up by infiltrated macrophages in TME, which then leads to enhancement of PD-L1 expression in macrophages and inhibition of T cell function, ultimately promoting HCC immune escape [147].

**Table 4:** Other TME stress-regulated ncRNAs and their roles during cancer progression

NcRNAs	Expression upon stress conditions	Regulatory mechanisms	Functions	Cancer types	References
lncRNA GOLGA2P10	Up-regulated under ER stress	PERK/ATF4/CHOP pathway	Regulate cancer cell apoptosis	Hepatocellular carcinoma	[145]
lncRNA MALAT1	Up-regulated under ER stress	IRE1/XBP1 and PERK/elF2α/ATF4 pathways	Promote cancer metastasis	Colorectal cancer	[146]
miR-23a-3p	Up-regulated under ER stress	N.A	Promote immune escape	Hepatocellular carcinoma	[147]
lncRNA HISLA	Up-regulated under acidosis	ERK/ELK1 signaling	Promote aerobic glycolysis and chemoresistance	Breast cancer	[148]

Oxidative stress results from the imbalance between the production of oxidative agents and antioxidants. Excessive free radicals such as reactive oxygen species (ROS) or reactive nitrogen species (RNS) often accumulate in cancer, leading to oxidative stress conditions [6]. Substantial miRNAs are reported to be regulated by oxidative stress via diverse mechanisms [13], whereas there is very limited literature discussing the exact mechanisms by which oxidative stress modulates lncRNA and circRNA expression. Considering that the increase of ROS and RNS can activate multiple signaling pathways such as MAPK, NF-κB, STAT3, PPARγ, or NRF2 to regulate the redox status of cells [6], these signaling pathways are also likely to exert regulatory effects on ncRNAs. Meanwhile, elevated ROS and RNS have been well-identified to trigger epigenetic reprogramming by modulating different chromatin-modifying enzymes [149–151], representing another potential regulatory mechanism for ncRNAs upon oxidative stress.

As a result of aerobic glycolysis, glucose in cancer cells is mainly processed into lactate, forming an acidic TME. Acidic TME strongly influences tumorigenesis [152]. A recent study by Chen et al. showed that lactate in the TME upregulated lncRNA HISLA in TAMs. Further mechanistic study uncovered that lactate activated ERK/ELK1 signaling in TAMs, which was proven to be responsible for the upregulation of HISLA. TAMs subsequently released extracellular vesicles containing HISLA to promote aerobic glycolysis and chemoresistance of breast cancer cells [148].

Overall, further investigations are warranted to elucidate the comprehensive regulatory networks of ncRNAs under various kinds of TME stress.

## Conclusions and perspectives

Over the course of tumor development, tumor cells are subjected to various adverse factors in TME: hypoxia, inflammation, nutrient deprivation, oxidative stress, acidosis, ER stress, and physical pressure. Under these TME stress conditions, a series of genomic alterations and adaptive responses are activated in tumor cells to support their continued growth, survival, and metastasis [2–6]. Given that TME plays a fundamental role during multiple stages of cancer development, future research will be aimed at deciphering the modes of action and in-depth molecular mechanisms of TME. Currently, as one of the most fast-emerging fields of research, ncRNAs have provided novel insights into the complexity of regulation under multiple stresses. Owing to its flexible structure for interaction and quick biogenesis nature, ncRNAs could be uniquely suited to provide a rapid, precise, and reversible response to major intrinsic changes and adverse environmental challenges. An increasing body of evidence has proved that various stresses in TME dramatically alter the expression levels of ncRNAs. These abnormally expressed ncRNAs participate in many biological processes of tumorigenesis.

Previous studies primarily centered around elucidating the biological functions and downstream effects of dysregulated ncRNAs in multiple cancer types, which have been extensively reviewed elsewhere [7, 8, 68, 153, 154]. However, there is still a lack of mechanistic studies to unravel how ncRNA levels are abnormally altered in cancer. Since ncRNA expression has been widely observed to be profoundly affected by TME stress conditions, the molecular mechanisms whereby TME stresses regulate ncRNA expression remain to be further clarified. As discussed throughout this review, we elaborate on the signal transduction pathways and epigenetic pathways through which HIFs, inflammatory factors, and nutrient deprivation in TME regulate ncRNAs, and highlight the multifaceted roles of TME stress-related ncRNAs in tumors. The mechanistic dissection of various TME stresses and their regulatory patterns on ncRNAs has advanced our understanding of tumor formation and progression. Nevertheless, more detailed mechanistic studies are still required, particularly in terms of other stress conditions like acidosis, oxidative stress, and ER stress. In addition to the well-appreciated transcriptional regulation and epigenetic modification, post-transcriptional regulation also profoundly impacts ncRNA biogenesis, stability, and biological functions. However, whether TME stresses affect post-transcriptional regulation remains largely elusive. Further, diverse post-translational modifications of proteins like histone, transcription factors, chromatin-modifying enzymes, and RNA polymerase II are known to be regulated by various stress stimuli [144, 155, 156], while their subsequent influences on ncRNA expression yet remain obscure. On the other hand, the current knowledge regarding the regulatory effects of TME stress principally focuses on miRNAs and lncRNAs, whereas emerging ncRNA species like circRNAs and piRNAs await investigation. Moreover, another important question is raised by the close integration of various types of TME stresses. Various stresses tend to coexist in tumor tissues. For instance, the occurrence of hypoxia often results in metabolic stress and ER stress [14, 16]. A hypoxic state and abnormal metabolic state induce the production of ROS, which aggravates oxidative stress [6]. This adds complexity and heterogeneity to the regulatory networks in vivo. Accordingly, it would be crucial to investigate how these stresses interact with each other and cooperate to influence ncRNA expression and cancer development. Additionally, it is interesting to further study the role (feedforward loop) of different ncRNAs in aggravating or inhibiting the TME stress. Lastly, to our best knowledge, the existing literature mainly focuses on discussing the roles of TME stress-responsive ncRNAs in regulating the cellular behavior of neoplastic cells, meanwhile, it is also fascinating to discuss their impact on different stromal cells, including endothelial cells, CAFs, as well as immune cells which are indispensable components of the TME, since this regulatory network may also indirectly contributes to tumor pathogenesis and progression.

Given the prominent pathological roles played by ncRNAs in cancer, novel classes of RNA-based therapeutics have shown potential to be clinically beneficial in the treatment of many diseases including cancer. For example, the antisense oligonucleotide and small interfering RNAs are employed to deplete lncRNA directly. Splice-switching oligonucleotide can be utilized to excise lncRNA exon that encodes necessary functional domains. Steric blocking oligonucleotides may interfere with the interaction of lncRNAs with their binding partners. These RNA-based methods present possibilities to modulate the functions of lncRNAs in diverse ways. Some of these drugs have been developed and are in clinical trials or in some cases approved for clinical use [157]. Furthermore, in light of our deeper understanding of how TME stress regulates numerous ncRNAs in the process of tumorigenesis and cancer progression, we propose that interfering with the regulatory network between TME stresses and ncRNAs, specifically by targeting key transcription factors, major signal transduction pathways, epigenetic pathways, or post-transcriptional modification could also be potentially feasible for cancer treatment. Finally, it is important to highlight that targeting different ncRNAs in combination with other drugs or inhibitors which can target TME stress shows great therapeutic benefit in cancer treatment. Particularly due to the feedback loops that frequently exist between TME-stress-regulated signaling pathways and ncRNAs, the combined therapy may display synergistic effects in promoting or inbihiting the regulatory function of TME stress on tumor development. In the study of Xiang et al., tumor suppressor miR-146b repletion and JAK inhibition exert greater inhibitory effects on breast cancer cell viability than either agent alone [76]. Another study of Li et al. reveals that interventions targeting circRNA cGGNBP2 provide potential auxiliary benefits for IL-6/STAT3-based treatments in ICC [81]. Currently, many drugs or inhibitors that can target TME-stress-regulated signaling pathways have been developed, such as acriflavine and belzutifan which target HIF [158, 159], siltuximab which targets IL-6 [160]. However, there are very limited investigations on the combined therapy of these inhibitors and ncRNAs, which deserves to be further studied.

It is hopeful that over the next decades, as the elaborate regulatory networks between TME and ncRNAs in cancer pathogenesis are described in greater detail and clarity, it may provide promising biomarker and therapeutic targets for cancer therapy.

## Supplementary Information

**Additional file 1:** **Figure S1.** Heatmaps showing differentially expressed ncRNAs in response to several TME stress. **a **Heatmap showing most upregulated and downregulated lncRNAs in OSCC cells cultured under normoxic and hypoxic conditions [30].** b **Differentially expressed lncRNAs in normoxic and hypoxic MCF10A cells [56]. **c** Most upregulated and downregulated lncRNAs (more than 10-fold) by various inflammatory stimuli (LPS, IL-1β, TNF-α) [86]. **d **Expression profiles of differentially regulated lncRNAs (over 2-fold change in expression) in three ESCC cell lines (KYSE30, KYSE180, KYSE450) treated with PBS or IFN-β [95]. **e** Heatmap showing top upregulated or downregulated lncRNAs in A2780s ovarian cancer cell line treated with recombinant CXCL14 protein or control [100]. **f** Differentially expressed lncRNAs in 786-O cells with or without glucose treatment [121].

## Data Availability

Not applicable.

## Abbreviations

AMPK: Adenosine monophosphate-activated protein kinase
AS: Alternative splicing
CAFs: Cancer-associated fibroblasts
CCL21: CC-chemokine ligand 21
ChIP: Chromatin immunoprecipitation
CHOP: C/EBP-homologous protein
CRC: Colorectal cancer
CXCL14: CXC-chemokine ligand 14
DNMT1: DNA methyltransferase 1
EMSA: Electrophoretic mobility shift assay
ER: Endoplasmic reticulum
ESCC: Esophageal squamous cell carcinoma
GBM: Glioblastoma
HCC: Hepatocellular carcinoma
HDACs: Histone deacetylases
HIFs: Hypoxia-inducible factors
HREs: Hypoxia response elements
HSPCs: Hematopoietic stem and progenitor cells
HuR: Human antigen R
ICC: Intrahepatic cholangiocarcinoma
IFNs: Interferons
ILs: Interleukins
JAK/STAT: Janus kinase-signal transducer and activator of transcription
KDMs: Histone lysine demethylases
lncRNAs: Long ncRNAs
MDSCs: Myeloid-derived suppressor cells
miRNAs: MicroRNAs
N.A.: Not available
ncRNAs: Noncoding RNAs
NF-κB: Nuclear Factor-kB
OSCC: Oral squamous cell carcinoma
PDAC: Pancreatic ductal adenocarcinoma
piRNAs: PIWI-interacting RNAs
PTEN: Phosphatase and tensin homolog
RBPs: RNA-binding proteins
RCC: Renal cell carcinoma
RNS: Reactive nitrogen species
ROS: Reactive oxygen species
siRNAs: Small interfering RNAs
sncRNAs: Small ncRNAs
snoRNAs: Small nucleolar RNAs
snRNA: Small nuclear RNAs
SP1: Specific protein 1
TAMs: Tumor-associated macrophages
TET2: Ten-eleven-translocation 2
TET3: Ten-eleven-translocation 3
TME: Tumor microenvironment
TNFs: Tumor necrosis factors
tsRNAs: Transfer RNA-derived small RNAs
UPR: Unfolded protein response
VHL: Von Hippel-Lindau protein
