# Supplementary material for: Noncoding RNAs as sensors of tumor microenvironmental stress
Source: J Exp Clin Cancer Res. 2022 Jul 16;41:224. doi: 10.1186/s13046-022-02433-y (PMC9288030; doi:10.1186/s13046-022-02433-y)
Supplement: Supplementary file 1 — Additional file 1: Figure S1. Heatmaps showing differentially expressed ncRNAs in response to several TME stress. a Heatmap showing most upregulated and downregulated lncRNAs in OSCC cells cultured under normoxic and hypoxic conditions [30]. b Differentially expressed lncRNAs in normoxic and hypoxic MCF10A cells [56]. c Most upregulated and downregulated lncRNAs (more than 10-fold) by various inflammatory stimuli (LPS, IL-1β, TNF-α) [86]. d Expression profiles of differentially regulated lncRNAs (over 2-fold change in expression) in three ESCC cell lines (KYSE30, KYSE180, KYSE450) treated with PBS or IFN-β [95]. e Heatmap showing top upregulated or downregulated lncRNAs in A2780s ovarian cancer cell line treated with recombinant CXCL14 protein or control [100]. f Differentially expressed lncRNAs in 786-O cells with or without glucose treatment [121]. [file 13046_2022_2433_MOESM1_ESM.docx]

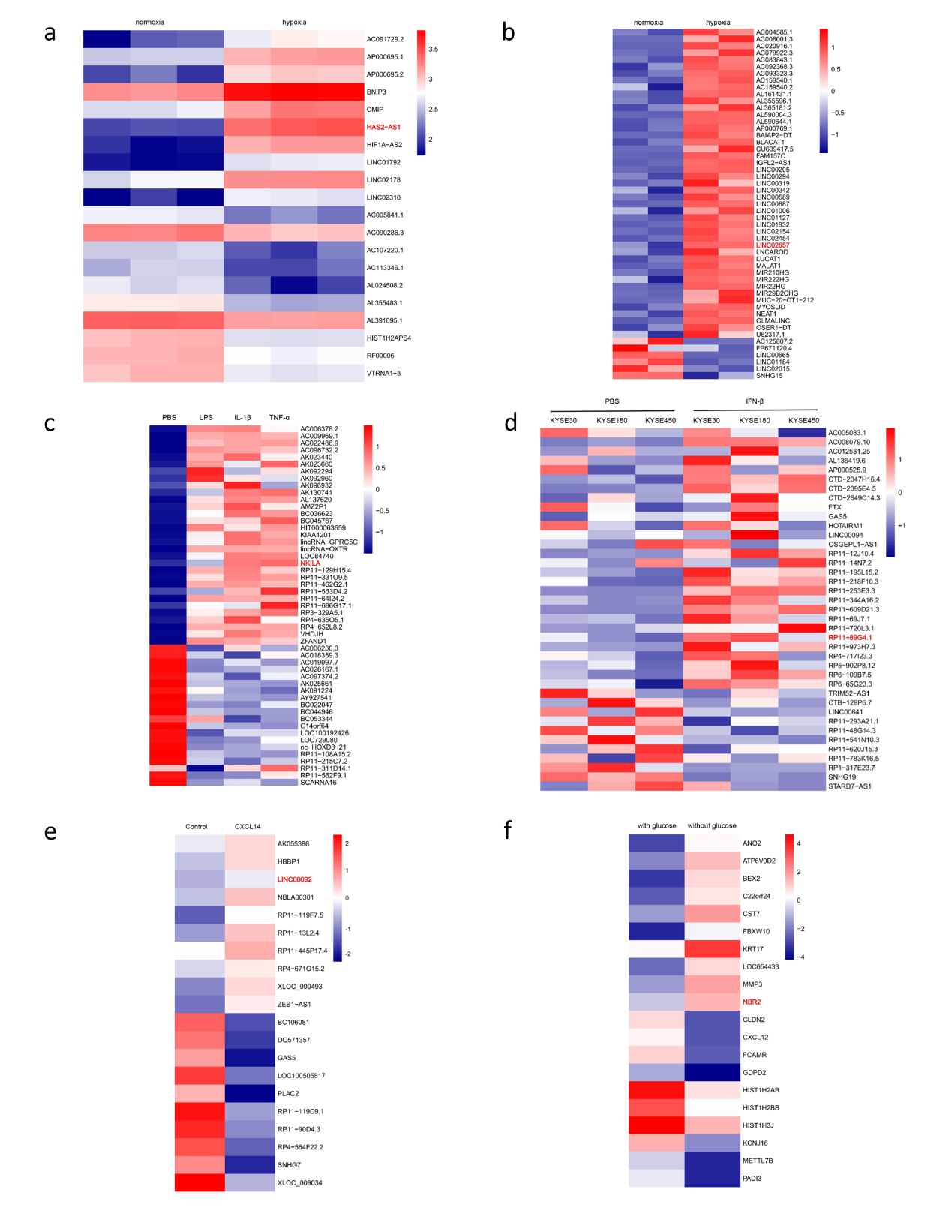


**Figure S1.** Heatmaps showing differentially expressed ncRNAs in response to several TME stress. **a** Heatmap showing most upregulated and downregulated lncRNAs in OSCC cells cultured under normoxic and hypoxic conditions [30]. **b** Differentially expressed lncRNAs in normoxic and hypoxic MCF10A cells [56]. **c** Most upregulated and downregulated lncRNAs (more than 10-fold) by various inflammatory stimuli (LPS, IL-1β, TNF-α) [86]. **d** Expression profiles of differentially regulated lncRNAs (over 2-fold change in expression) in three ESCC cell lines (KYSE30, KYSE180, KYSE450) treated with PBS or IFN-β [95]. **e** Heatmap showing top upregulated or downregulated lncRNAs in A2780s ovarian cancer cell line treated with recombinant CXCL14 protein or control [100]. **f** Differentially expressed lncRNAs in 786-O cells with or without glucose treatment [121].
